# Energy-Effective Power Control Algorithm with Mobility Prediction for 5G Heterogeneous Cloud Radio Access Network

**DOI:** 10.3390/s18092904

**Published:** 2018-09-01

**Authors:** Hyebin Park, Yujin Lim

**Affiliations:** Department of IT Engineering, Sookmyung Women’s University, Seoul 04310, Korea; hb0390@sookmyung.ac.kr

**Keywords:** 5G, heterogeneous cloud radio access network, vehicular mobility, remote radio head switching operation

## Abstract

In 5G networks, heterogeneous cloud radio access network (H-CRAN) is considered a promising future architecture to minimize energy consumption and efficiently allocate resources. However, with the increase in the number of users, studies are performed to overcome the energy consumption problems. In this study, we propose a power control algorithm with mobility prediction to provide a high-energy efficiency for 5G H-CRAN. In particular, the proposed algorithm predicts UE mobility in vehicular mobility scenarios and performs remote radio head (RRH) switching operations based on % prediction results. We formulate an optimization problem to maximize the energy efficiency while satisfying the outage probability requirement. We then propose an RRH switching operation based on Markov mobility prediction and optimize the transmission power based on a gradient method. Simulation results demonstrate the improved energy efficiency compared with those of existing RRH switching-operation algorithms.

## 1. Introduction

With the rapid increase in mobile data traffic, cellular networks are expected to provide high-capacity services to users. In order to satisfy the requirements, cellular networks have been studied and many network architectures have been developed. In traditional network architectures, one base station (BS) is deployed in one cell structure. In order to improve the original system bandwidth and performance, many architectures have been studied for future 5G cellular networks. As a larger data capacity is provided, the energy consumption has become an important issue. The data capacity and energy consumption exhibit a “trade-off” relationship. In high-capacity services, the energy consumption from the BS side corresponds to approximately 75% of the total energy consumption in cellular networks [1]. Considering the increasing energy consumption, studies are performed for an effective and minimized energy consumption.

Heterogeneous network (HetNet), cloud radio access network (C-RAN), and heterogeneous CRAN (H-CRAN) are representative architectures. First, in HetNet, one macrocell and multi-small-cells are overlapped and deployed densely in hot spots to improve spectrum efficiency. The small cells consist of multi-picocells or -femtocells, which cover a smaller range than a macrocell. Small cells closely associate with users so that the system can provide a higher data rate to users and reduce the transmission power. Second, in order to minimize the energy consumption, the BS structure is separated in the C-RAN architecture. A traditional BS consists of two parts: base band unit (BBU), which processes signals, and remote radio head (RRH), which serves as a radio-frequency transceiver. In C-RAN, BSs are divided into the two parts to reduce energy consumption. BBUs are centralized as a BBU pool and RRHs are distributed and connected to the BBU pool through fronthaul links. Third, the H-CRAN architecture has been studied to utilize the advantages of both HetNet and C-RAN. In H-CRAN, pico- or femto-RRHs are connected to the BBU pool through fronthaul links and a macro BS (MBS) is connected to the BBU pool through a backhaul link [2]. H-CRAN provides services by two parts: the MBS provides administration of voice service, while the RRHs mainly provide high-data-rate services. RRHs serve only as radio-frequency transceivers, while the BBU pool manages resources with a centralized architecture, so that it is possible to efficiently allocate resources in H-CRAN.

With the implementation of the architectures in network systems, studies considering user mobility have been performed to provide personalized services. Users usually move in certain patterns in their daily lives (i.e., the places they visit can be regarded as certain patterns). In particular, users who use vehicles have more predictable patterns as it is difficult to suddenly change speed or direction. In addition, in urban areas, vehicles tend to move more consistently in patterns owing to temporal and spatial factors. There are various rules such as traffic lights that vehicles have to follow while moving in certain patterns. Vehicles generally move only along designated roads, which makes their mobility patterns more consistent. They can be provided with information about blocked road sections during periods of traffic congestion. Therefore, vehicular mobility patterns in urban areas can be introduced for various applications.

In this study, we propose a joint power control algorithm for an efficient energy consumption consisting of three parts. By patterning mobilities of vehicle users, we predict mobility patterns based on a Markov prediction algorithm. We consider vehicular mobilities in urban environments for mobility prediction, owing to the consistent patterns in urban scenarios. We refer to vehicles as “user equipment” (UE). Based on the UE mobility prediction, we control the RRH switching operation and transmission power to maximize the energy efficiency.

The remainder of this paper is organized as follows. In Section 2, we introduce related studies. In Section 3, the system model and problem are formulated. The energy-effective power control algorithm is proposed in Section 4. In Section 5, simulation results and discussion are presented. The conclusions of this study are presented in Section 6.

## 2. Related Studies 

With the development of high-data-rate services, the energy consumption issue attracts increasing attention. In 5G networks, more BSs have been installed to provide high-quality services to the large number of UEs. To improve energy efficiency in densely deployed BS environments, energy-efficient optimization algorithms are proposed [3,4]. In [3], an optimization framework is developed in mmWave-based ultradense networks. In ultradense networks, most users tend to associate with MBS because MBS services higher data capacity. So it proposes a load-aware user association to redistribute the concentrated users. In order to update transmit power and associate users in an optimal way, an iterative gradient method is proposed. In [4], a subchannel and power allocation algorithm is developed for combined HetNet with a visible light communication (VLC) system. It transforms the energy-efficient resource allocation problem into a convex optimization problem and solves the problem with an alternative direction method of multipliers.

BS switching operation is a popular approach to overcome the energy efficiency and consumption problems. In order to overcome the problem with guaranteed quality-of-service (QoS) requirements, various approaches have been considered in recent studies. For HetNet, many studies have been performed to overcome the energy consumption problem [5,6,7]. In [5], a decentralized sleep scheme with user association and resource allocation is proposed to minimize energy consumption. It solves the problem with a modified matching algorithm and voting-based algorithm. After a signal-to-interference-plus-noise-ratio (SINR)-based user association, small BSs vote for another BS that has the lowest traffic load around it. The proposed algorithm selects a small BS that has the lowest ratio of served users to the vote score. In [6], a QoS-aware topology management with transfer learning is developed. It uses QoS information from spectrum assignment as knowledge, with management transfer from the spectrum assignment knowledge to user association. A BS switching strategy with a clustering technique has been reported in [7]. It decomposes the problem into user association, BS clustering, and BS switching operation, with BS clustering into several groups and switching-operation control by clusters.

C-RAN has a coexisting structure consisting of a centralized BBU pool and distributed RRHs, providing efficient resource allocation and data processing. In order to address the energy consumption problem, RRH activation strategies have been proposed for optimization using the Lagrangian theory [8,9,10]. In [8], an RRH activation algorithm considering resource allocation is implemented. It considers and minimizes outage probability, which is the rate of users who do not receive the required minimum. In [9], a joint RRH activation and beamforming algorithm is proposed, transferring the nonconvex problem to a concave–convex fractional program using a weighted minimum mean-square-error method. It employs the mixed $n_{1}/n_{p}$ norm in sparse beamforming. In [10], an RRH activation algorithm considering data requirements of users is proposed. It formulates the user association as a knapsack problem to satisfy user requirements. It then organizes an active RRH set and deactivates RRHs that are not a part of the active RRH set.

H-CRAN is another future architecture combining HetNet and C-RAN. In [11], a joint resource assignment and power allocation algorithm design is proposed. In order to maximize the energy efficiency, an enhanced soft fractional frequency reuse is used to characterize user association. It formulates the resource assignment and power allocation as a nonconvex function derived with the Lagrange dual-decomposition method. In [12], a joint RRH switching algorithm design with user association and resource allocation is proposed. It formulates the problem considering a limited fronthaul capacity. It solves the problem with RRH switching-operation activation with a greedy algorithm and subchannel assignment with a Lagrangian multiplier.

In addition, there is a need for location-based services to provide high-quality services to users. In order to address the issues, user mobility prediction has been studied [13,14,15]. In [13], an improved Markov algorithm is proposed to predict the user’s next location from the trajectory. It predicts the user’s next location using the history of the user’s trajectory consisting of time-interval-representative location pairs. In [14], forecasting of a mobility model with a neural network is reported and analyzed in comparison with real mobility data; the mobility model is employed in an urban mobile ad hoc network and a back-propagation neural network is used for time-series prediction. In [15], predictions based on Markov models for vehicle routes in urban settings are developed; they consider not only dynamic traffic conditions in the urban area that affect the route selection, but also mobile taxi contexts, and separates roads into several segments. As this characterizes correlations between routes of vehicles and traffic conditions, trace-driven mobility patterns are extracted.

As the number of UEs increases continuously, more BSs have been deployed to satisfy the increasing data demands. The BS switching operations have been actively underway to address energy issues in densely deployed BS environments. However, previous works mainly focus on defining the problem to maximize the energy efficiency through optimization techniques. In the realistic network environment, traffic conditions continuously change according to user mobility. Thus, BS switching operation is needed considering user mobility to provide energy-efficient, high-quality services in time-varying traffic conditions. In addition, the previous works mainly have interest in the pedestrians’ mobility. However, we consider the users’ mobility in vehicles, which have the consistent patterns and higher speed than pedestrians. With consideration of vehicular mobility, we propose RRH switching operation in the H-CRAN architecture as the BS switching operation.

To improve energy efficiency in H-CRAN with high mobility environments, we propose that an energy-efficient power algorithm with mobility prediction provides RRH switching operation based on the predicted mobility. The RRH switching operation controls the activation and deactivation of RRHs according to the traffic. If more RRHs are activated when there are reductions in UEs according to the traffic or mobility in the cell, RRHs are switched off to increase the energy efficiency. Conversely, if there are UEs that cannot receive service owing to an increasing traffic in the cell, RRHs are switched on. With user mobility characteristics in the process of RRH switching, the focus is on reducing the number of users serviced by the suboptimal RRHs. We also present low-complexity power allocations that can be applied even in high-mobility environments.

## 3. System Model and Problem Formulation

**Figure 1:**
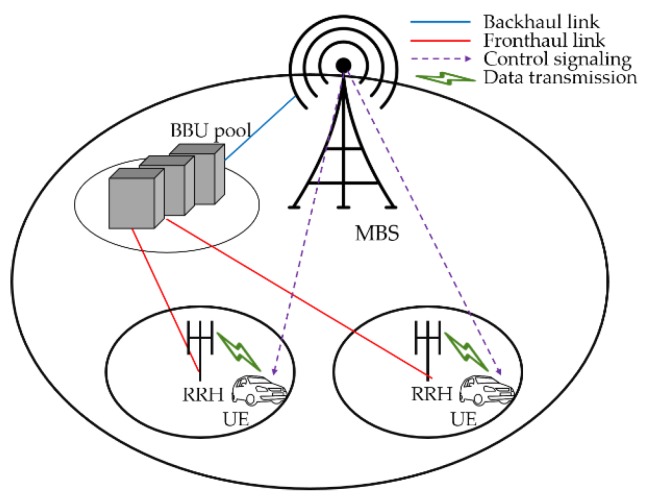
Architecture of H-CRAN.

We consider a single-cell architecture in H-CRAN consisting of a BBU pool connected to a single MBS and set of RRHs, as shown in Figure 1. The MBS provides the voice services and transmits control signaling to UEs; therefore, we consider a power consumption model without the power consumption of the MBS. We denote the set of RRHs and UEs in a cell as S and U, respectively, and define $a_{s}$ as an indicator of the binary activation of an RRH s, with values of 0 for a deactivated RRH and 1 for an activated RRH.

The power consumption model includes the powers consumed by RRHs, fronthaul links, and backhaul links. The power consumption of an RRH can be expressed as:

(1) $$P_{RRH,s}=a_{s}\left(\Phi_{RRH}+∆s\overset{U}{\underset{u=1}{\sum }}b_{u}^{s}p_{t}^{s}\right)+\left(1-a_{s}\right)\Phi_{Sleep},\;\;\;\;s\in \left\{1,\cdots ,S\right\},\;u\in \left\{1,\cdots ,\;U\right\},$$

where $\Phi_{RRH}$ is the circuit power of RRH s, which is activated, $b_{u}^{s}$ is the binary association indicator of UE u in RRH s, with values of 1 for an associated UE and 0 for a nonassociated UE, $p_{t}^{s}$ is the non-negative transmission power of RRH s, $\Phi_{Sleep}$ is the power consumption of the deactivated RRH s, and ∆s is the slope of the load-dependent power model of the RRH. The power consumption of a fronthaul link reported in [8] can be expressed as:

(2) $$P_{fh,s}=a_{s}\left(\Phi_{fh}+\beta t_{s}\right),$$

where $\Phi_{fh}$ is the constant power consumption from the fronthaul transceiver and switch, β is the power consumption per bit/s at the fronthaul link, and $t_{s}$ is the total traffic on the fronthaul link, which is associated with RRH s. The power consumption model of the backhaul link $P_{bh}$ is also reported in [16], so that we set the power consumption of the backhaul link as a constant value. We express the total power consumption model of a single cell in the H-CRAN architecture as:

(3) $$P=\overset{S}{\underset{s=1}{\sum }}\left(P_{RRH,s}+P_{fh,s}\right)+P_{bh}.$$

The SINR to UE u from RRH s can be expressed as:

(4) $$SINR_{s}^{u}=\frac{p_{t}^{s}g_{u}^{s}}{\sum_{i\neq s}p_{t}^{i}g_{u}^{i}+N_{0}},$$

where $g_{u}^{s}$ is the channel gain between RRH s and UE u, and $N_{0}$ is the noise power spectral density. The achievable data rate from RRH s can be expressed as:

(5) $$R_{s}=B\overset{U}{\underset{u=1}{\sum }}\log_{2}\left(1+SINR_{u}^{s}\right),$$

where B is the total channel bandwidth of the system. Therefore, the sum of achievable data rates from the RRHs is:

(6) $$R=\overset{S}{\underset{s=1}{\sum }}a_{s}R_{s}.$$

The total energy efficiency (EE) of the system can be defined as a ratio of the sum of achievable data rates to the total power consumption:

(7) $$EE=\frac{R}{P}.$$

Our main goal is to maximize the total energy efficiency subject to a certain QoS constraint of UEs. The considered QoS constraint is outage probability, which is a ratio of the number of UEs that are not provided with a certain SINR threshold $SINR_{th}$ to the total number of UEs. We denote the outage probability as $P_{outage}$. The energy-efficiency maximization problem can be formulated as:

(8) $$\begin{array}{c} \underset{s,p_{t}^{s}}{\max}\;EE\left(p_{t}^{s}\right)\\ s.t.C1:\;\overset{U}{\underset{u=1}{\sum }}a_{s}b_{u}^{s}=1\;\forall u,\\ C2:\;a_{s}\overset{U}{\underset{u=1}{\sum }}b_{u}^{s}p_{t}^{s}\leq p_{t}^{max}\;\forall s,\\ C3:P_{outage}\leq P_{outage}^{max} \end{array}$$

where ∀u∈U,s∈S, $a=\left[a_{1},\;\cdots ,a_{S}\right]$, $b=\left[b_{1}^{1},\cdots ,\;b_{U}^{S}\right],$ and $p=\left[p_{t}^{1},\cdots ,p_{t}^{S}\right]$. C1 implies that each user accesses only one RRH at a time, C2 ensures that the transmission power is not larger than the upper bound (the maximum transmission power is denoted as $p_{t}^{max}$), and C3 implies that the outage probability cannot exceed the maximum outage probability $P_{outage}^{max}$ [17].

## 4. Proposed Algorithm 

In order to solve Equation (8), we introduce the power control algorithm consisting of the following two procedures. First, an RRH switching operation is performed based on the predicted UEs’ mobilities. If UEs are not connected with any RRHs or UEs can be provided with better SINRs than those by the currently serving RRHs, the UEs are reassociated based on the SINRs. Second, an optimal transmission power of the RRHs is calculated to maximize the energy efficiency.

### 4.1. RRH Switching Operation and SINR-Based User Association

We propose an RRH switching operation with mobility prediction and SINR-based user association. The mobility prediction is based on a k-order Markov algorithm consisting of the following steps [13]:

1. The trajectory $T_{d}$ is organized for each UE consisting of a list of serving RRHs at regular intervals, where $T_{d}$ is the d^th^ day’s history (1≤d≤D); the trajectory $T_{d}$ is then divided into time intervals t.
2. The RRH that appears most frequently in each interval is selected as a representative RRH and a representative history is developed. The latest k representative RRHs are considered, indicated as $pattern_{k}$, where 1≤k≤t, and a $pattern_{s}$ is created by combining with all RRHs, where s∈S.
3. The probability of appearance in the next interval $P\left(pattern_{s}\right)$ is calculated for each $pattern_{s}$:
  (9) $$P\left(pattern_{s}\right)=S^{\frac{\theta_{s}}{\left|T_{d}\right|}}\times \frac{N_{pattern_{s}}}{N_{pattern_{k}}},$$
  where $\left|T_{d}\right|$ is the size of the trajectory, $\theta_{s}$ is the number of occurrences of RRH s in the trajectory $T_{d}$, $N_{pattern_{s}}$ is the number of $pattern_{s}$’s occurrences in the history, and $N_{pattern_{k}}$ is the number of $pattern_{k}$’s occurrences in the history.
4. The RRH s with the highest probability $P\left(pattern_{s}\right)$ is used as the next serving RRH s˜ for the UE u; the predicted RRH $\tilde{s}$ corresponds to RRH s.

Based on the results of the mobility prediction, the RRH switching operation is performed. In order to increase the proportion of UEs served at the optimal RRHs, RRH s with the largest $\rho_{\tilde{s}}$ is deactivated among the activated RRHs, where $\rho_{\tilde{s}}$ represents an influence on UEs served when RRH s is switched off. If an RRH s with a large $\rho_{\tilde{s}}$ is switched off, fewer UEs are served by a suboptimal RRH as fewer UEs are reassociated. It can be expressed as:

(10) $$\rho_{\tilde{s}}=\frac{\lambda_{\tilde{s}}\;}{\sum_{u=1}^{U}b_{u}^{\tilde{s}}},$$

where $\lambda_{\tilde{s}}$ is the inverse of the minimum resource block load in RRH $\tilde{s}$, expressed as:

(11) $$\lambda_{\tilde{s}}={\left(\frac{\sum_{u=1}^{U}b_{u}^{\tilde{s}}\gamma_{u}}{RB}\right)}^{-1},$$

where $\gamma_{u}$ is the minimum number of resource blocks to provide the data rate requirement to UE u [18]; $\gamma_{u}$ can be calculated as:

(12) $$\gamma_{u}=\lceil \frac{d_{u}\;}{B\log_{2}\left(1+SINR_{u}^{\tilde{s}}\right)}\rceil ,$$

where $d_{u}$ is the minimum required data rate of UE u. After the RRH switching operation, a reassociation is performed. The proposed algorithm is summarized in Algorithm 1.
**Algorithm 1:** RRH switching operation with mobility prediction and SINR-based user association.1: calculate EE using Equation (7); set $EE_{curr}=EE$ and $EE_{prev}=0$2: calculate $P\left(pattern_{s}\right)$ for all RRH s using Equation (9)3: calculate $\rho_{s}$ for all RRH s using Equation (10)4: while $EE_{curr}>EE_{prev}$ do5:  set $EE_{prev}=EE_{curr}$6:  if $\overset{U}{\underset{u=1}{\sum }}\gamma_{u}>RB,\;\forall s$ then7:    deactivate the activated RRH s that has the largest $\rho_{\tilde{s}}$8:  else9:    activate the deactivated RRH s that has the smallest $\rho_{\tilde{s}}$10:  end if11:  do user association for all UE u with an active RRH providing the highest SINR12:  calculate $EE_{curr}$ using Equation (7)13: end while14: solve Equation (8) using Algorithm 215: calculate EE using Equation (7)


### 4.2. Optimization of Transmission Power Based on a Gradient Method

In Algorithm 1, by the RRH switching operation according to the traffic of the cell, the interference on the UEs from the other RRHs was minimized. In this section, we use the gradient method to optimize the transmission power for an increased total-energy efficiency. The optimization of the transmission power is performed by the following steps within the upper and lower bounds of the transmission power.

In order to find the optimal transmission power $p_{t}^{*}$, we iteratively calculate the transmission power at which the derivative of Equation (8) is zero. We denote the derivative of $p_{t}^{s}$ in Equation (8) as $\eta \left(p_{t}^{s}\right)$:

(13) $$\begin{array}{ll} \eta \left(p_{t}^{s}\right) & =\frac{\partial EE\left(p_{t}^{s}\right)}{\partial p_{t}^{s}}\\ & =\frac{B\sum_{u\in U\;}\left(\frac{1}{I_{u}^{i}\left(p_{t}^{s}\right)+N_{0}}-\frac{p_{t}^{s}}{{\left(I_{u}^{i}\left(p_{t}^{i}\right)+N_{0}\right)}^{2}}\right)}{\ln2\left(1+SINR_{u}^{s}\right)\cdot P}-\frac{\sum_{u\in U}\ln\left(1+SINR_{u}^{s}\right)}{\ln2{\left(P\right)}^{2}}\;, \end{array}$$

where $I_{u}^{i}\left(p_{t}^{i}\right)$ is the interference on UE u from other RRH i≠s and $I_{u}^{i}\left(p_{t}^{i}\right)=\underset{i\neq s}{\sum }p_{t}^{i}g_{u}^{i}$. We use a positive step size α for gradual convergence and multiply it by the derivative $\eta \left(p_{t}^{s}\right)$. We update $p_{t}^{s}$ by:

(14) $$p_{t}^{s}=p_{t}^{s}+\alpha \cdot \eta \left(p_{t}^{s}\right).$$

After updating the transmission power, we calculate the energy efficiency and compare it to that before the update. If the energy efficiency has increased, we set the updated transmission power as the optimal transmission power $p_{t}^{*}$. This method is repeated up to the maximum number of iterations K or until the difference in energy efficiency is smaller than a given convergence condition ϵ; further details are presented in Algorithm 2. The step size α is set to be smaller at the iterations, enabling a faster convergence. The proposed optimization method is summarized in Algorithm 2.
**Algorithm 2:** Optimization of the transmission power based on the gradient method.1: set the maximum iteration number K and convergence condition ϵ2: set the positive step size α3: set $p_{t}^{k}$ = $p_{t}^{s}$, $p_{t}^{k+1}=0$, k=1, $p_{t}^{*}=p_{t}^{k}$ calculate $EE\left(p_{t}^{k}\right)$ using Equation (8)4: for 1≤k≤K do5:  set k=k+16:  $p_{t}^{k+1}=p_{t}^{k}+\alpha \cdot \eta^{k}\left(p_{t}^{k}\right)$7:  if $EE\left(p_{t}^{k+1}\right)>EE\left(p_{t}^{k}\right)$ then8:    $p_{t}^{*}$ = $p_{t}^{k+1}$.9:  else if $\;EE\left(p_{t}^{k+1}\right)-EE\left(p_{t}^{k}\right)\leq \epsilon$ then10:    break11:  end if12:  set α=α/k13: end for


## 5. Results and Discussion

A single-cell H-CRAN environment is considered; the simulation parameters are summarized in Table 1. We set the parameters according to the 3GPP specifications [19]. In the single macrocell with an intercell distance of 1 km, ten RRHs and variable UEs are randomly distributed. The UE number |U| is dynamically set by the simulation setting. Following the 3GPP specifications [20], we consider Rayleigh fading and log-normal shadowing. We also consider the small-cell path loss model: 140.7 + 36.7logd(km), where d is the distance between the RRH and UE, and noise figure of 9 dB.

The datasets used in the simulation are composed of taxi mobility trace data acquired in San Francisco [21]. In addition, another dataset with taxi mobility data acquired in Beijing is used to apply the algorithms to different mobilities [22]. We used the datasets to organize the history records at various speeds, denoted as 1x, 2x, and 4x. In addition, two distribution scenarios are considered, where $d_{u}$ is set to have Gaussian or uniform distribution.

We compare the proposed algorithm with other algorithms. First, the proposed algorithm is basically an online algorithm because of predicting mobility. So, the offline version algorithm without mobility prediction is used to analyze performance of the proposed algorithm. We denote the online algorithm with mobility prediction as ‘Proposed (online)’ and the offline algorithm as ‘Proposed (offline)’. The difference between the online algorithm and the offline algorithm is in Equation (10) for switching RRH. In the online algorithm, Equation (10) uses the predicted RRH $\tilde{s}$, but the offline algorithm uses the current RRH s. In other words, the offline algorithm performs RRH switching without the process of predicting user mobility, and the selection of RRHs is calculated based on current associated RRHs, not on predicted results. Second, the full-activation (FA) algorithm is used in the comparison to evaluate the baseline performance. Third, the algorithm in [4] in HetNet is used, which has decentralized small BSs. Fourth, for a comparison with the same network environment, the algorithm in [12] is used. They are denoted as ‘activation algorithm in HetNet (A-HN)’ and ‘activation algorithm H-CRAN (A-HC)’ in the following figures, respectively.

**Figure 2:**
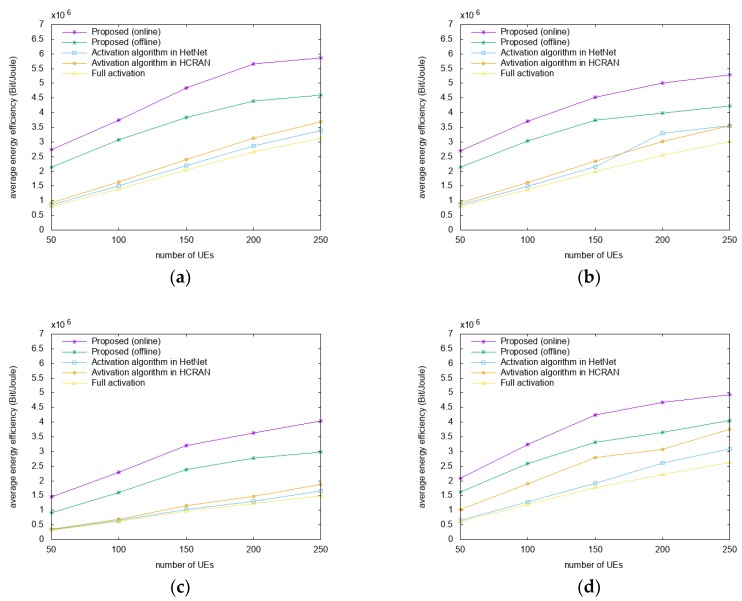
Comparison of energy efficiencies. (**a**) San Francisco and (**c**) Beijing datasets with Gaussian distributions; (**b**) San Francisco and (**d**) Beijing datasets with uniform distributions.

In Figure 2, the energy efficiencies are compared for different datasets and distribution scenarios as a function of the number of users. For the scenarios presented in Figure 2a,c with Gaussian distributions, the proposed algorithm has a higher performance by approximately 42%–68% and 58%–77% than A-HN and A-HC, respectively. In the scenarios presented in Figure 2b,d with uniform distribution, the proposed algorithm has a higher performance by approximately 32–68% and 37%–68% compared to A-HN and A-HC, respectively. Compared to the FA algorithm in all of the scenarios, energy efficiency gains of approximately 43%–70% are achieved by the proposed algorithm. Compared to the offline algorithm, the proposed online algorithm outperforms approximately 17%–22% and 17%–36% in San Francisco and Beijing datasets, respectively. Also, the offline algorithm has a higher performance by about 15%–59% and 15%–56% compared to A-HN and A-HC, respectively.

The proposed algorithm firstly switches off the RRH servicing the smaller number of users or the smaller amount of resource blocks, compared with other RRHs. Therefore, through the reassociation process, the number of users who are serviced from the suboptimal RRHs is reduced. This, in turn, works to enable users to receive higher SINRs and increase the energy efficiency of the networks. Besides, the proposed algorithm increases energy efficiency by predicting user mobility, allowing users to receive higher SINRs. In other words, the proposed algorithm switches the RRHs to enable the users to receive service from the optimal RRHs through mobility prediction before the user moves. This is achieved as A-HN and A-HC control RRHs considering energy consumption, while the proposed algorithm controls the RRHs while maximizing the SINR.

**Figure 3:**
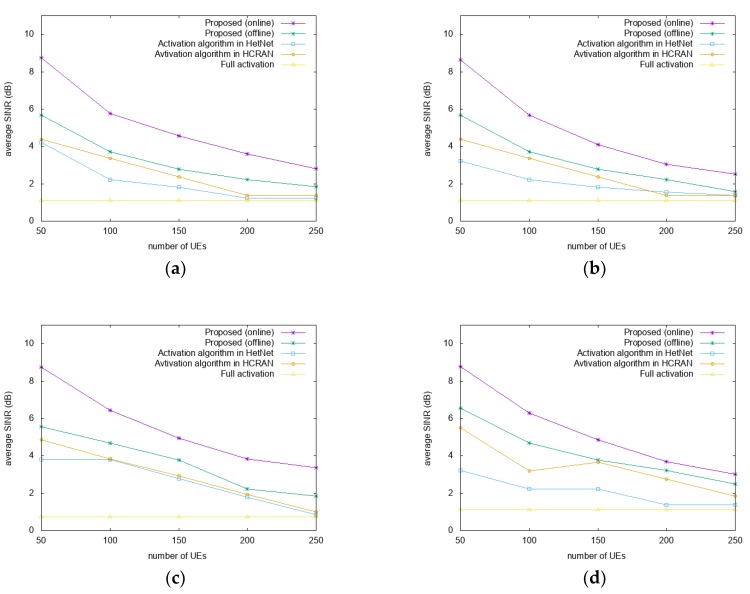
Comparison of the average SINRs. (**a**) San Francisco and (**c**) Beijing datasets with Gaussian distributions; (**b**) San Francisco and (**d**) Beijing datasets with uniform distributions.

Figure 3 shows a comparison of the average SINRs for different datasets and distribution scenarios as a function of the number of users. In Figure 3a,c, where Gaussian distributions are used, the proposed algorithm has an SINR approximately 51%–61% and 41%–74% higher than those of A-HN and A-HC, respectively. In Figure 3b,d, where uniform distributions are used, the SINR of the proposed algorithm is 45%–62% and 54%–63% higher than those of A-HN and A-HC, respectively. Considering all of the scenarios, the proposed algorithm has a better performance by approximately 56%–91% compared to FA. Compared to the offline algorithm, the proposed algorithm performs approximately 26%–39% and 12%–44% higher at San Francisco and Beijing datasets, respectively. Also, the offline algorithm has a 13%–45% and 9%–38% higher SINR than A-HN and A-HC, respectively, in Figure 3.

With the increase in the number of users in the simulation, the average SINR tends to decrease owing to the increased number of active RRHs. This occurs as, upon the increase in the number of active RRHs with the increase in the number of UEs, the SINR determined by the interference among RRHs is reduced. In the proposed algorithm, the RRH which services the small number of users or small amount of resource blocks is preferred to turn off the switch. In the reassociation through the RRH switching operation, the proposed algorithm reduces the associations between UEs and RRHs that correspond to suboptimal SINRs, which explains the higher SINR than those of the other algorithms.

**Figure 4:**
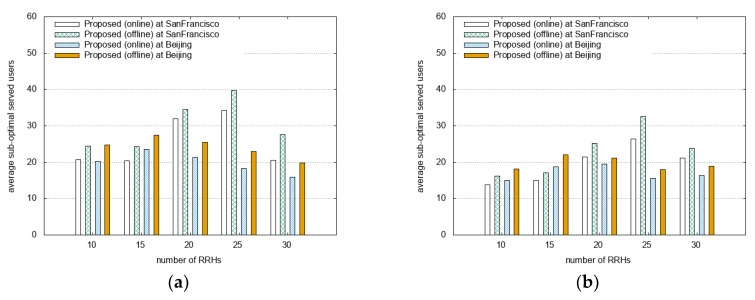

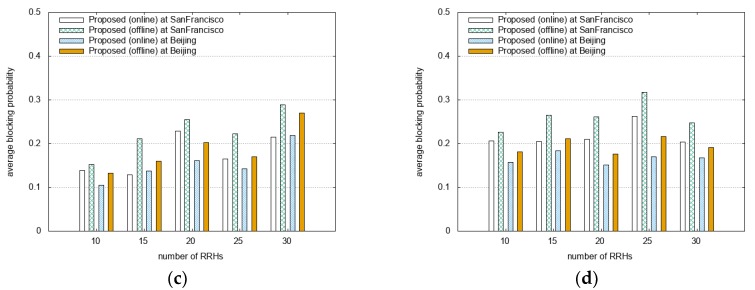
Comparison of the online and offline algorithm with 500 UEs. (**a**) The number of suboptimal served users with Gaussian distribution and (**b**) with uniform distribution; (**c**) blocking probability with Gaussian distribution and (**d**) with uniform distribution.

Figure 4 shows a comparison of the number of suboptimal served users and blocking probability for different datasets and distribution scenarios as a function of the number of RRHs. Our goal is reducing the number of suboptimal served users and decreasing the blocking probability. The number of suboptimal served users are the number of users who are reassociated with suboptimal RRHs because current serving RRHs have been switched OFF. In the reassociation process, the user can be rejected if the amount of requested resource blocks exceeds the available resource blocks of suboptimal RRH. The blocking probability is the ratio of the number of rejected users at reassociation to the number of suboptimal served users. For the scenarios presented in Figure 4a,b with Gaussian and uniform distribution, the online algorithm shows approximately 8%–33% fewer sub-optimal served users compared to the offline algorithm. In Figure 4c,d, the online algorithm gets approximately 9%–64% lower blocking probability than the offline algorithm. We consider vehicular mobility environment that leads to more frequent handovers and mobility patterns than pedestrians. In the highly mobile environment, the online algorithm brings better performance, because RRH is switched based on the number of servicing users and the number of minimum requested resource blocks on current served RRHs (offline) or predicted RRHs (online).

**Figure 5:**
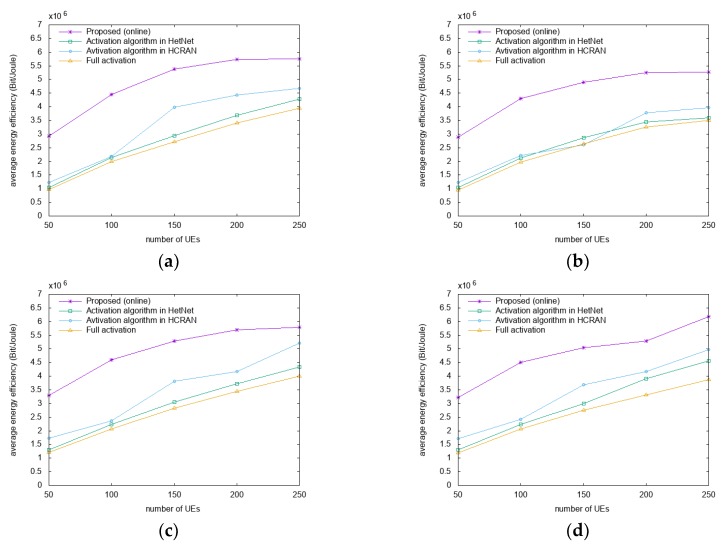
Comparison of the energy efficiencies. (**a**) 2x and (**c)** 4x speeds with Gaussian distributions; (**b**) 2x and (**d**) 4x speeds with uniform distributions.

Figure 5 shows a comparison of the energy efficiencies for the San Francisco dataset for different speeds and distribution scenarios as a function of the number of users. In Figure 5a,b where the speeds are 2x, the proposed algorithm has a higher energy efficiency by approximately 18–64% and 24%–64% compared to A-HN and A-HC, respectively. In Figure 5c,d, where the speeds are 4x, the proposed algorithm has a higher energy efficiency by approximately 10%–60% and 19%–59% compared to A-HN and A-HC, respectively. In all of the scenarios, the proposed algorithm has a higher energy efficiency by approximately 30%–67% compared to FA.

Gains in average energy efficiency are observed with the increase in the mobility speed, as the mobility with a higher speed has a more consistent pattern. A mobility with consistent patterns is more likely to be predicted, so that the proposed algorithm can provide a high-quality service based on prediction. However, the other algorithms in the comparison do not consider the mobility, but only the current environments, and perform the switching operation, which explains the higher performance of the proposed algorithm in high-speed scenarios, compared to A-HN and A-HC.

**Figure 6:**
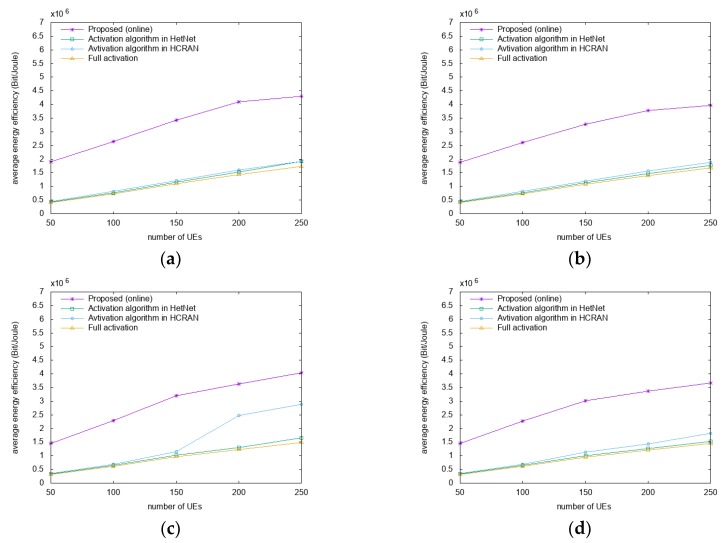
Comparison of the energy efficiencies in environments with 15 RRHs. (**a**) San Francisco and (**c**) Beijing datasets with Gaussian distributions; (**b**) San Francisco and (**d**) Beijing datasets with uniform distributions.

Figure 6 shows a comparison of the energy efficiencies in environments with 15 RRHs for different datasets and distribution scenarios as a function of the number of users. Many RRHs offer higher-quality data services; however, there are also interference problems. Therefore, it is important to provide service by optimal RRHs in a highly interfering environment. Simulations were performed to evaluate the changes in energy efficiency when more RRHs were deployed. In Figure 6a,c with Gaussian distributions, the proposed algorithm has a higher energy efficiency by approximately 55%–77% and 52%–77% compared to A-HN and A-HC, respectively. In Figure 6b,d with uniform distributions, the proposed algorithm has a higher energy efficiency by approximately 28%–77% and 50%–77% compared to A-HN and A-HC, respectively. In all of the scenarios, the proposed algorithm has a higher energy efficiency by approximately 31–67% compared to the FA algorithm. The proposed algorithm is better than the other algorithms as it enables users to receive services by optimal RRHs among the various RRHs.

In order to evaluate the performance of the proposed algorithm, comparisons were performed with FA and two other algorithms. The proposed algorithm exhibited the best performance in terms of energy efficiency, as the number of UEs receiving services from optimal RRHs was increased. As the SINR received by UEs increases, the system can consume less energy while providing the same data rate, yielding a higher energy efficiency. In order to demonstrate this analysis, we performed SINR comparisons, where the proposed algorithm was better than the other algorithms. In addition, we showed that the proposed algorithm performs well in environments with increased number of RRHs and UE speed.

## 6. Conclusions

We proposed a power control algorithm to maximize the energy efficiency in the H-CRAN architecture. We formulated the maximization problem of the energy efficiency using the outage probability. We designed the RRH switching operation based on mobility prediction using the Markov method. We considered the service of UEs and use of resource blocks to maximize the number of UEs serviced by optimal RRHs while performing the RRH switching operation. The algorithm finds the optimal transmission power with the gradient method to maximize the energy efficiency. The simulation results demonstrated that the proposed algorithm provided the best energy efficiency and SINR among the compared algorithms.

In the UE mobility prediction, there is an issue in terms of the number of employed history trajectories. By increasing the number of trajectories, the prediction accuracy could increase; however, storage problems emerge owing to the large number of UEs. In addition, predictions using the existing Markov method are less accurate in movements with irregular patterns. Therefore, it is required to develop a technology to adapt the use of trajectories and accordingly compensate irregular patterns. In environments with a variety of mobility, traditional network architectures often generate unnecessary handovers. In addition, as the need for location-based services increases, traffic is being frequently generated to provide data to satisfy demands for location-based services. Therefore, it is necessary to perform further studies combined with new future architecture like F-RAN (Fog Radio Access Networks) [23] that solves such problems by increasing the use of endpoint devices. It can relieve the burden of fronthaul and BBU pool and manage resources efficiently from changing mobility.

## Figures and Tables

**Table 1:** Simulation parameters.

Parameter	Notation	Value
Channel bandwidth	B	100 MHz
Noise power spectral density	$N_{0}$	−174 (dBm/Hz)
Requirement threshold of SINR	$SINR_{th}$	0 (dB)
Maximum outage probability	$P_{outage}^{max}$	0.05
Number of resource blocks	RB	50
Maximum transmission power of RRH	$p_{t}^{max,\;s}$	30 dBm
Constant power of active RRH	$\Phi_{RRH}$	6.8 W
Power of sleep RRH	$\Phi_{Sleep}$	4.3 W
Slope of RRH	Δs	4.0
Constant power of backhaul link	$P_{bh}$	13.25 $W^{3}$
Power consumption per bit/s of fronthaul link	β	0.83 W
Constant power of fronthaul link	$\Phi_{fh}$	13 W
Minimum required data rate of UE	$d_{u}$	512, 1024, 1536, 2048, 2560 kbps
Maximum iteration number	K	100
Positive step size	α	0.001
